# *Morinda citrifolia* Linn. Reduces Parasite Load and Modulates Cytokines and Extracellular Matrix Proteins in C57BL/6 Mice Infected with *Leishmania (Leishmania) amazonensis*

**DOI:** 10.1371/journal.pntd.0004900

**Published:** 2016-08-31

**Authors:** Fernando Almeida-Souza, Flávia de Oliveira Cardoso, Bruno Vinicius da Conceição Souza, Tânia Zaverucha do Valle, Joicy Cortez de Sá, Iara dos Santos da Silva Oliveira, Celeste da Silva Freitas de Souza, Carla Junqueira Moragas Tellis, Maria do Socorro dos Santos Chagas, Maria Dutra Behrens, Ana Lúcia Abreu-Silva, Kátia da Silva Calabrese

**Affiliations:** 1 Laboratório de Imunomodulação e Protozoologia, Instituto Oswaldo Cruz-Fiocruz, Rio de Janeiro, Brazil; 2 Departamento de Patologia, Universidade Estadual do Maranhão, São Luís, Brazil; 3 Departamento de Produtos Naturais, Farmanguinhos-Fiocruz, Rio de Janeiro, Brazil; University of Texas Medical Branch, UNITED STATES

## Abstract

The absence of an effective vaccine and the debilitating chemotherapy for Leishmaniasis demonstrate the need for developing alternative treatments. Several studies conducted with *Morinda citrifolia* have shown various biological activities, including antileishmanial activity, however its mechanisms of action are unknown. This study aimed to analyze the in vivo activity of *M*. *citrifolia* fruit juice (Noni) against *Leishmania (Leishmania) amazonensis* in C57BL/6 mice. *M*. *citrifolia* fruit juice from the Brazilian Amazon has shown the same constitution of other juices produced around the world and liquid chromatography–mass spectrometry analysis identified five compounds: deacetylasperulosidic acid, asperulosidic acid, rutin, nonioside B and nonioside C. Daily intragastric treatment with Noni was carried out after 55 days of *L*. *(L*.*) amazonensis* infection in C57BL/6 mice. Parasitic loads, cytokine and extracellular protein matrix expressions of the lesion site were analyzed by qPCR. Histopathology of the lesion site, lymph nodes and liver were performed to evaluate the inflammatory processes. Cytokines and biochemical parameters of toxicity from sera were also evaluated. The Noni treatment at 500 mg.kg^-1^.day^-1^ for 60 days decreased the lesion size and parasitic load in the footpad infected with *L*. *(L*.*) amazonensis*. The site of infection also showed decreased inflammatory infiltrates and decreased cytokine expressions for IL-12, TNF-α, TGF-β and IL-10. On the other hand, Noni treatment enhanced the extracellular matrix protein expressions of collagen IV, fibronectin and laminin in the infected footpad as well collagen I and II, fibronectin and laminin in the mock-infected footpads. No toxicity was observed at the end of treatment. These data show the efficacy of Noni treatment.

## Author Summary

Leishmaniasis is a complex of diseases caused by parasites of the *Leishmania* genus, which affects thousands of people around the world. The parasite lives within the cells and the disease manifests itself in different ways, one of them is wound-like lesions on the skin that do not heal. The treatment, with a medicament discovered in 1912, causes several side effects, its intramuscular administration is painful and it is given daily over a long period of time. These characteristics show the need for new alternatives for leishmaniasis treatment justifying works like this. The *Morinda citrifolia* is a plant native to the Polynesian islands and has a fruit commonly known as Noni. Noni has been analyzed for various targets such as anticancer, anti-inflammatory and antimicrobial effects; however, the antileishmanial has not yet been fully evaluated. This work proves that Noni treatment can promote a fast clinical cure in mice with leishmaniasis by decreasing parasite number, acting on the immune system and repairing skin components.

## Introduction

Leishmaniasis is one of the seventeen neglected diseases prioritized by the World Health Organization. Although most cases of neglected diseases are in underdeveloped countries, leishmaniasis is spreading worldwide [1]. The infection caused by *Leishmania* parasites may remain asymptomatic or evolve to a symptomatic form that can vary from a cutaneous to a visceral form of the disease, the latter of which can be lethal if left untreated [2].

As there is no vaccine against leishmaniasis yet, infected people are treated with antileishmaniasis drugs and control still depends on programs focusing on the vector and reservoir hosts [1, 3]. There are a limited number of drugs for the treatment of leishmaniasis and the pentavalent antimonials are the most common [3]. However, antimonials can cause severe adverse effects, such as vomiting, nausea, anorexia, myalgia, abdominal pain, headache, arthralgia, and lethargy, due to their accumulation in the tissues [1]. Until now, efforts to reduce the toxicity of antileishmaniasis drugs have been unsuccessful, which reinforces the need for new antileishmanial drugs. Therefore, protocols that could provide an alternative therapy, reduce dosages, treatment duration and adverse effects for leishmaniasis, would be welcome.

*Morinda citrifolia* Linn. is a small plant native to Southeast Asia. It is commonly known as Noni and is one of the most significant resources of traditional medicine in S.E. Asian countries. The efficacy of Noni in the treatment of pain and inflammatory reactions [4] as well as its antimicrobial activity [5] has been demonstrated in various studies. Recently, morindicone and morinthone, isolated from the stem of *M*. *citrifolia*, were shown to have activity, in vitro, against *Leishmania (L*.*) major* [6].

In order to demonstrate the antileishmanial activity of *M*. *citrifolia*, our group has been using the fruit juice of this plant in *in vitro* assays with *Leishmania (L*.*) infantum* promastigotes and intracellular amastigotes. Our previous results showed cytoplasmic vacuolization, lipid inclusion, increased exocytosis activity and autophagosome-like vesicles in *L*. *(L*.*) infantum* promastigotes treated with *M*. *citrifolia* fruit juice. Cytotoxicity assay with J774.G8 macrophages showed that *M*. *citrifolia* fruit juice was not toxic to these cells up to 1000μg.mL^-1^; however, when intracellular amastigotes were evaluated by light microscopy, macrophages showed vacuoles with probable remains of intracellular parasites [7]. Based on these results, the aim of the present study was to evaluate the antileishmanial activity of *M*. *citrifolia* fruit juice under in vivo conditions, using C57BL/6 mice subcutaneously infected with *L*. *(L*.*) amazonensis*.

## Methods

### Plant material

*Morinda citrifolia* fruits were collected in São Luiz (S2°31 W44°16), a municipality in the Brazilian Amazon, located 24m above sea level. Fully ripe fruits, with a translucent exocarp, were picked in the rainy season, from April to November 2011. The material was properly identified by Ana Maria Maciel Leite and the voucher specimen number 2000346 was deposited at the Herbarium Professora Rosa Mochel at the Universidade Estadual do Maranhão. Fruits were washed with sterilized distilled water, dried at 25°C and placed in sterile glass bottles for 3 days to drain off the extract. The juice extract, called Noni, from *M*. *citrifolia* fruit was centrifuged twice at 4000 rpm for 15 minutes; the supernatant was lyophilized and stored at -20°C. Noni was dissolved in PBS immediately before use in the *in vivo* experiments.

### Liquid chromatography–mass spectrometry analysis (LCMS)

Lyophilized noni was dissolved in methanol to 5mg.mL^-1^. The LC Shimadzu Nexera UFLC was coupled to an ion trap Bruker Amazon. Analyses were performed at ambient temperature in a 100mm x 2.1mm x 2.6μm Kinetex C18 gravity column, equipped with an 8 mm x 4 mm, 5μm guard column. The mobile phase consisted of water containing 0.1% formic acid (eluent A) and acetonitrile (eluent B). The gradient of B was as follows: in 5.5 min from 5% to 25%, from 7.0 to 8.5 min up to 100% B, held at 100% for 1.5 min, then 100% to 5% in 1 min, and finally held at 5% for 2 min. The flow rate was 0.3 mL/min and the injection volume was 1 μL. Other specifications were as described in the literature [8].

### Animals

Female C57BL/6 mice 4-6-weeks old were obtained from Centro de Criação de Animais de Laboratório (CECAL/FIOCRUZ) and maintained under pathogen-free conditions, controlled temperature and food and water *ad libitum*.

### Ethics statement

All experiments with animals were conducted in accordance with the guidelines for experimental procedures of the Conselho Nacional de Controle de Experimentação Animal (CONCEA) and approved by Comissão de Ética no Uso de Animais from Fundação Oswaldo Cruz (CEUA-FIOCRUZ), identification number LW72/12.

### Parasites and infection

The *L*. *(L*.*) amazonensis* (MHOM/BR/1976/MA-76) obtained from a human case of diffuse infection and characterized by isoenzyme [9] and lectin techniques [10] was maintained in the laboratory by successive passages in BALB/c mice. Prior to infection, parasites were isolated from a non-ulcerated nodular lesion in the footpad and amastigote viability was checked with erythrosine B by light microscopy. 10^4^ amastigote forms were inoculated subcutaneously into the right footpad of C57BL/6 mice.

### Experimental procedures

Initially, an 8-week pilot treatment protocol, with two different concentrations of Noni (250 and 500mg.kg^-1^), was carried out to determine the dose of Noni to be used in the posterior analyses. The daily treatment was carried out with 100μL of Noni by gavage. A group of non-treated infected mice was maintained as control. Lesion thickness was evaluated weekly in order to choose the most efficient drug concentration.

Treatment protocol was performed with 5 groups of 10 animals, as follows: infected and treated (100μL of Noni 500mg.kg^-1^ by gavage, daily); infected and control drug-treated (Glucantime 20mg.kg^-1^ by intramuscular injection, twice a week); infected and mock-treated (100μL of PBS by gavage, daily); mock-infected and treated (100μL of Noni 500mg.kg^-1^ by gavage, daily); and normal (mock-infected and mock-treated). Treatment started 55 days after infection for all groups. Lesion kinetics was evaluated weekly by a caliper rule, in comparison to the non-infected contralateral footpad and expressed as lesion thickness. After 30 and 60 days of treatment animals were euthanized, blood was collected to obtain serum and tissue fragments from footpad, draining lymph nodes and liver were excised for posterior analyses.

### Parasite load by real time PCR

DNA from the footpad and draining lymph nodes of 3 animals per group was extracted following a standard phenol/chloroform protocol [11]. DNA concentration was quantified in a NanoDrop 2000c spectrophotometer (ThermoScientific). Parasite load was estimated by real time PCR performed in Applied Biosystems Step One Plus equipment, using Fast SYBR Green Master Mix. Primers were target for the parasite kDNA and mouse *β*-actin was used as an endogenous control (S1 Table).

### Histopathology

Skin, lymph nodes and liver fragments were fixed in 10% buffered formalin and routinely processed for paraffin embedding. Tissue sections (5μm thick) were stained with Hematoxylin-Eosin, Gomori trichrome and Picrosirius red. Tissues were observed under a light microscope and polarized light was used to observe the collagen fibers.

### Cytokine and extracellular matrix protein gene expression at the lesion site by RT-PCR

After euthanasia, skin fragments of infected footpads from 3 mice of each group were collected. Total RNA was extracted using TRIZOL reagent (Invitrogen, Karlsruhe, Germany) following the manufacturer’s instructions. cDNA synthesis was performed with 1μg of total RNA using a iScript cDNA Synthesis kit (Bio-Rad Laboratories, Hercules, CA) according to the manufacturer’s recommendations. Primers targeting the genes IL-4, IL-10, IL-12, TNF-*α*, IFN-*γ*, TGF-*β*, iNOS, Laminin, Fibronectin and Collagens I, III and IV were designed using the Primer Express software version 3.0 (Applied Biosystems, 2004), and manufactured by Invitrogen (Supplementary Data 1). Real Time PCR assays were performed using Power SYBR Green Master Mix and the relative quantification (2^-ΔΔCT^) method was applied, using the mouse RPLP0 gene (large ribosomal protein, P0) as the endogenous control. Results were analyzed with the StepOne Software v2.3 (Applied Biosystems).

### Quantification of cytokine production by ELISA

A pool of sera obtained from the blood of five mice per group was used for cytokine quantification of IL-4, IL-10, IL-12, TNF-*α*, IFN*γ* (BD Bioscience) and TGF-*β* (R&D System) following the manufacturer's specifications.

### Toxicity analysis parameters

Clinical signs of toxicity, such as piloerection, diarrhea, salivation, convulsions or changes in mobility, respiration rate or muscle tone, were observed during the treatments. Levels of alanine transaminase (ALT), aspartate transaminase (AST), alkaline phosphatase (ALP), total protein, direct bilirubin, indirect bilirubin, total bilirubin, albumin, globulin, urea and creatinine were analyzed in sera pools from mice treated for 60 days in Ciba Corning equipment. At necropsies, stomach and gut mucosa were macroscopically evaluated for abnormal findings. Animal weight was measured on an analytical balance after 30 and 60 days of treatment.

### Statistical analysis

The values were expressed as mean ± S.D. The results were analyzed statistically by Analysis of Variance (ANOVA) followed by Bonferroni’s post-test. The analyses were performed with the software GraphPad Prism 5.0.4. Differences were considered significant when p<0.05.

## Results

### Liquid chromatography–mass spectrometry analysis of Noni

**Fig 1:**
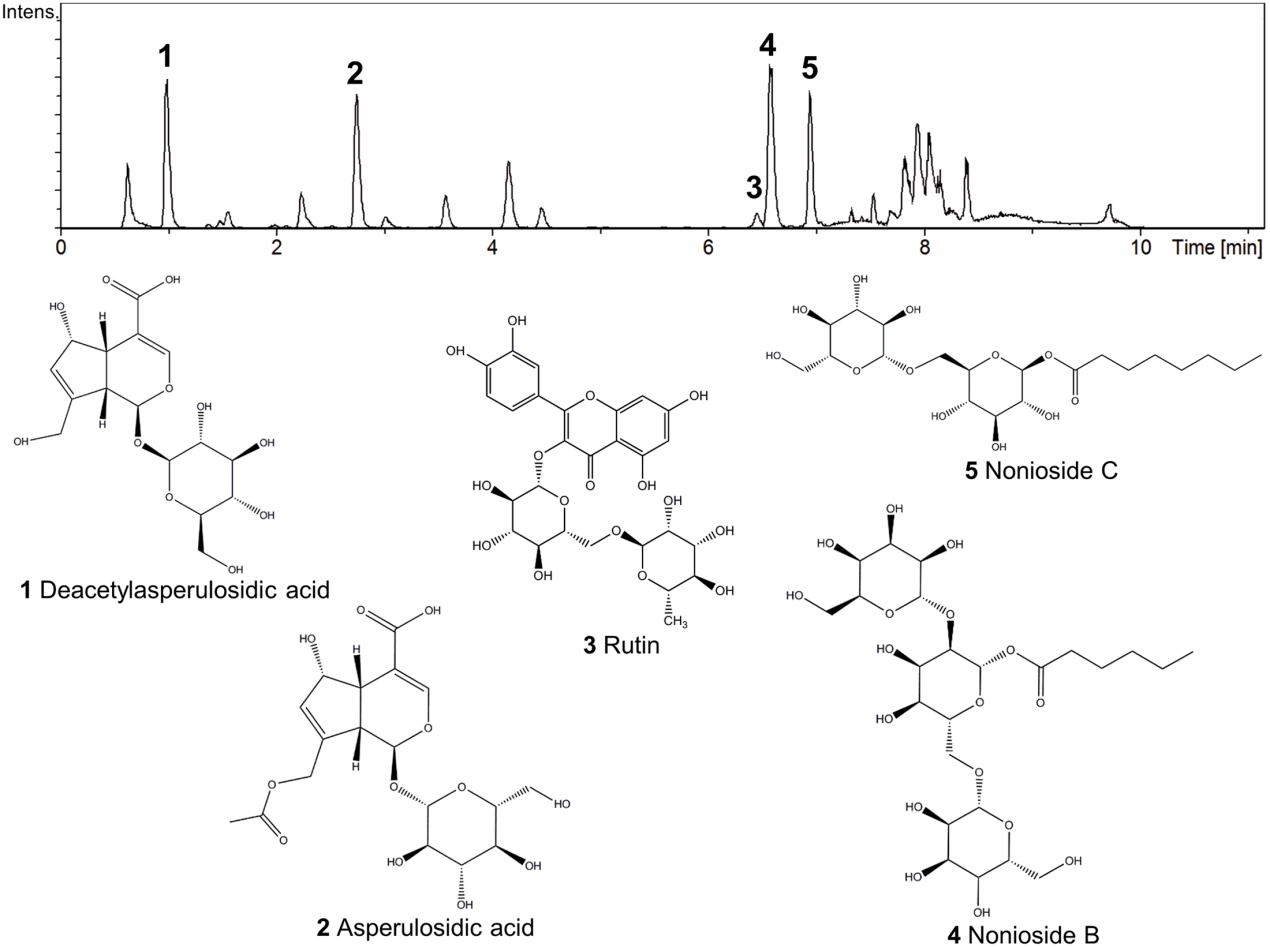
Liquid chromatography–mass spectrometry analysis of *Morinda citrifolia* fruit juice, Noni. (1–5) Chromatograms of compounds (m/z) identified in Noni: deacetylasperulosidic acid (389), asperulosidic acid (431), rutin (610), nonioside B (629) and nonioside C (467).

According to the selective ions and elution order obtained from the Liquid Chromatography–Mass spectrometry analysis and compared with references in the literature [8], five compounds were identified: deacetylasperulosidic acid (**1**), asperulosidic acid (**2**), rutin (**3**), nonioside B (**4**) and nonioside C (**5**) (Fig 1). The extract ion chromatograms (m/z) of these compounds were respectively 389, 431, 609, 629 and 467.

### Noni treatment decreases the lesion size growth and parasitic load

**Fig 2:**
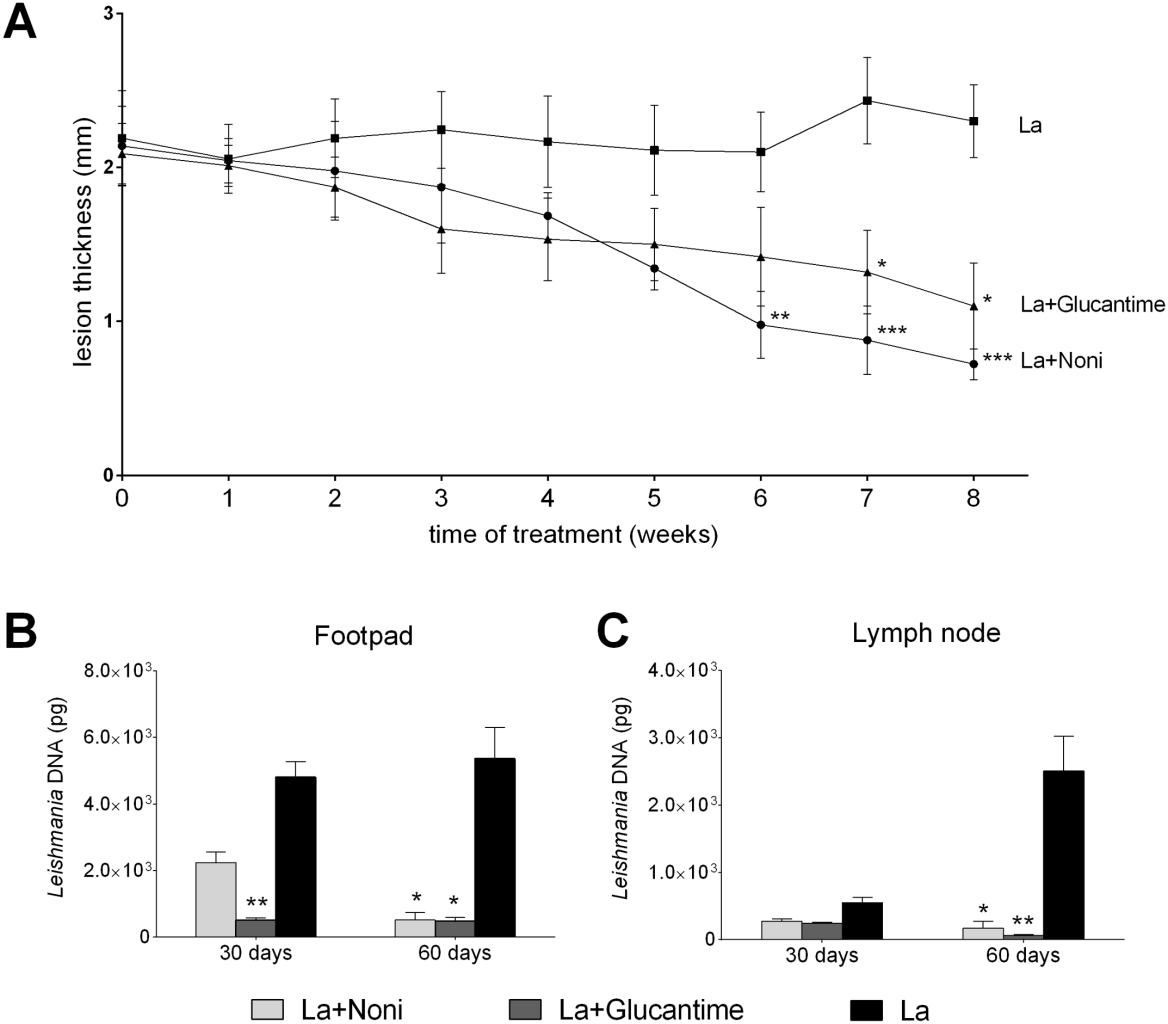
Activity of *Morinda citrifolia* fruit juice (Noni) treatment in C57BL/6 mice infected with *Leishmania (L*.*) amazonensis*. (A) Kinetic of lesion of infected footpads treated with Noni (500mg.kg^-1^.day^-1^) or Glucantime (20mg.kg^-1^.twice a week^-1^). (B-C) Parasite loads in footpad and draining lymph node after 30 and 60 days of Noni treatment. Data represent mean ± SD of two independent experiments realized at least in triplicate. *p<0.05, **p<0.01, ***p<0.001 when compared with La group by two-way ANOVA and Bonferroni’s post-test. La+Noni: group infected and treated with Noni; La+Glucantime: group infected and treated with Glucantime; La: group infected and mock-treated; Normal: mock-infected and mock-treated group.

The pilot protocol showed that Noni at 500mg.kg^-1^ could significantly reduce lesion growth from the fourth week of treatment. Therefore, the dosage of 500mg.kg^-1^ was chosen for subsequent protocols. In this protocol, the treatment was able to significantly reduce lesion size as of the sixth week, when compared with the infected non-treated group (Fig 2A). The control drug, Glucantime 20mg.kg^-1^, was also able to decrease lesion size, showing no statistical difference with Noni treatment.

After 30 days of Noni treatment there was no change but after 60 days the parasite loads in the footpad and draining lymph node had significantly decreased in comparison to the non-treated control, corroborating with the results of the lesion kinetics (Fig 2B and 2C). On the other hand, Glucantime was able to reduce parasite loads after 30 days of treatment for the footpad and after 60 days for the lymph node.

### Noni decreases inflammation of *L*. *(L*.*) amazonensis* infected mice

**Fig 3:**
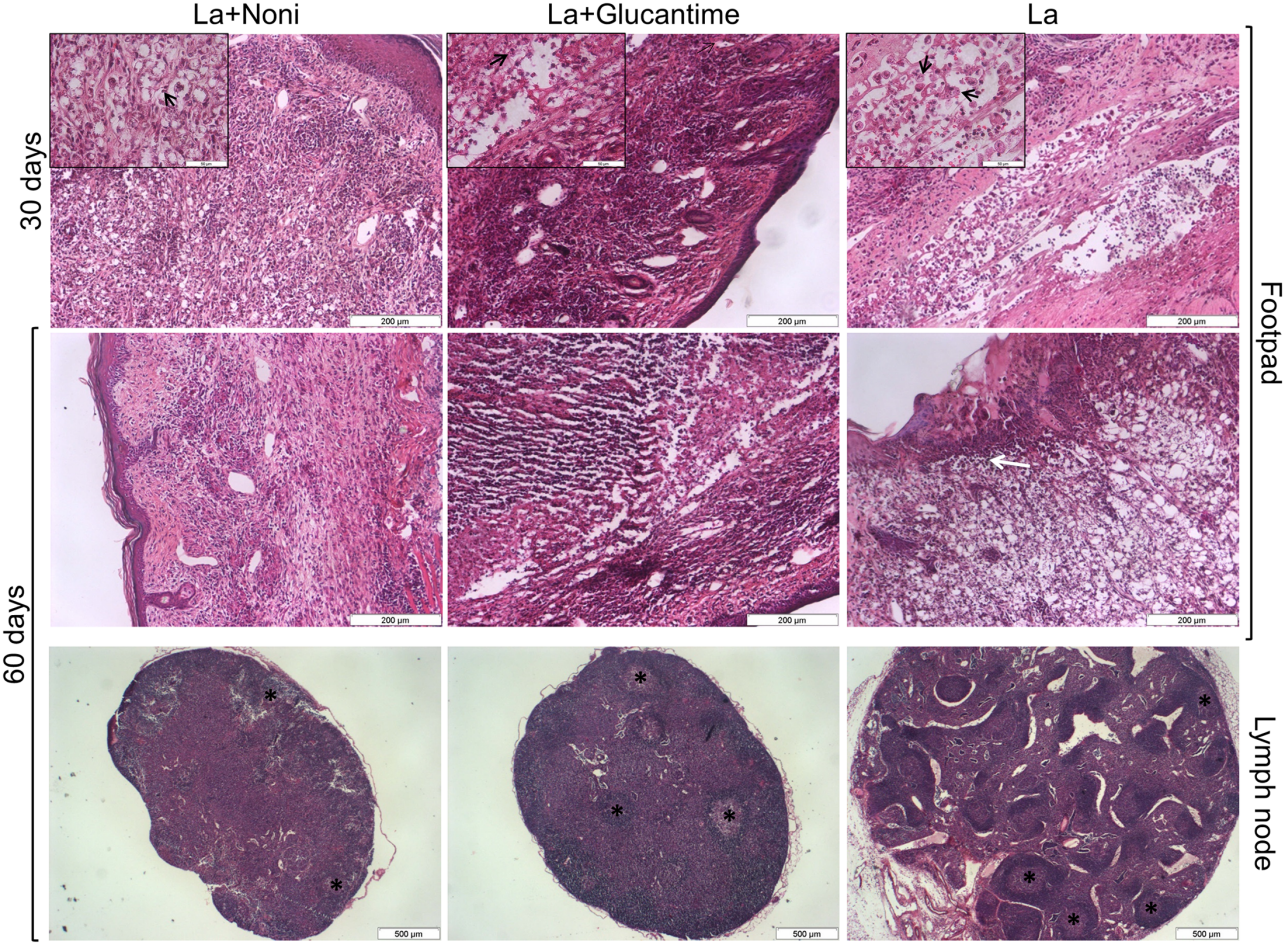
Histopathological analysis of skin and lymph nodes of C57BL/6 mice footpads infected with *Leishmania (L*.*) amazonensis* and treated with *Morinda citrifolia* fruit juice, Noni. Noni group treated at 500mg.kg^-1^.day^-1^ and Glucantime group treated at 20mg.kg^-1^.twice a week^-1^; both treatments were for 60 days. At 30 days: amastigotes within macrophages (arrows in inserts) and inflammatory infiltration in dermis of all groups. At 60 days: inflammatory infiltration decreased in the Noni group, and absence of amastigotes in the Noni and Glucantime groups. La group with intense inflammatory infiltration of macrophages with parasites at the lesion site. In lymph nodes, there was a decrease of the lymphoid nodules (asterisks) hyperplasia in Noni and Glucantime groups. Images representative of two experiments realized in triplicate. Hematoxylin-eosin.

Histopathological analysis of the lesion site of mock-treated mice showed inflammatory infiltrates composed of parasitized macrophages 30 and 60 days after infection (Fig 3). In the former, the infiltrated area had increased and the number of infected macrophages enhanced. Furthermore, a large area of necrosis and lesion ulcerations was observed. Also at 30 days after infection the draining lymph node presented hyperplasia of the cortical region. Noni treatment at 30 and 60 days reduced the parasite loads and inflammatory infiltrate. Remarkable tissue remodeling at the lesion site and depletion of the number of blast cells in the lymph node were observed after 60 days of treatment. Also, at that time, no parasites were found in the Glucantime-treated mice at the lesion site. A reduction of the inflammatory infiltrate was also noted in the skin as well as the reestablishment of the normal histopathological pattern of the lymph node.

**Fig 4:**
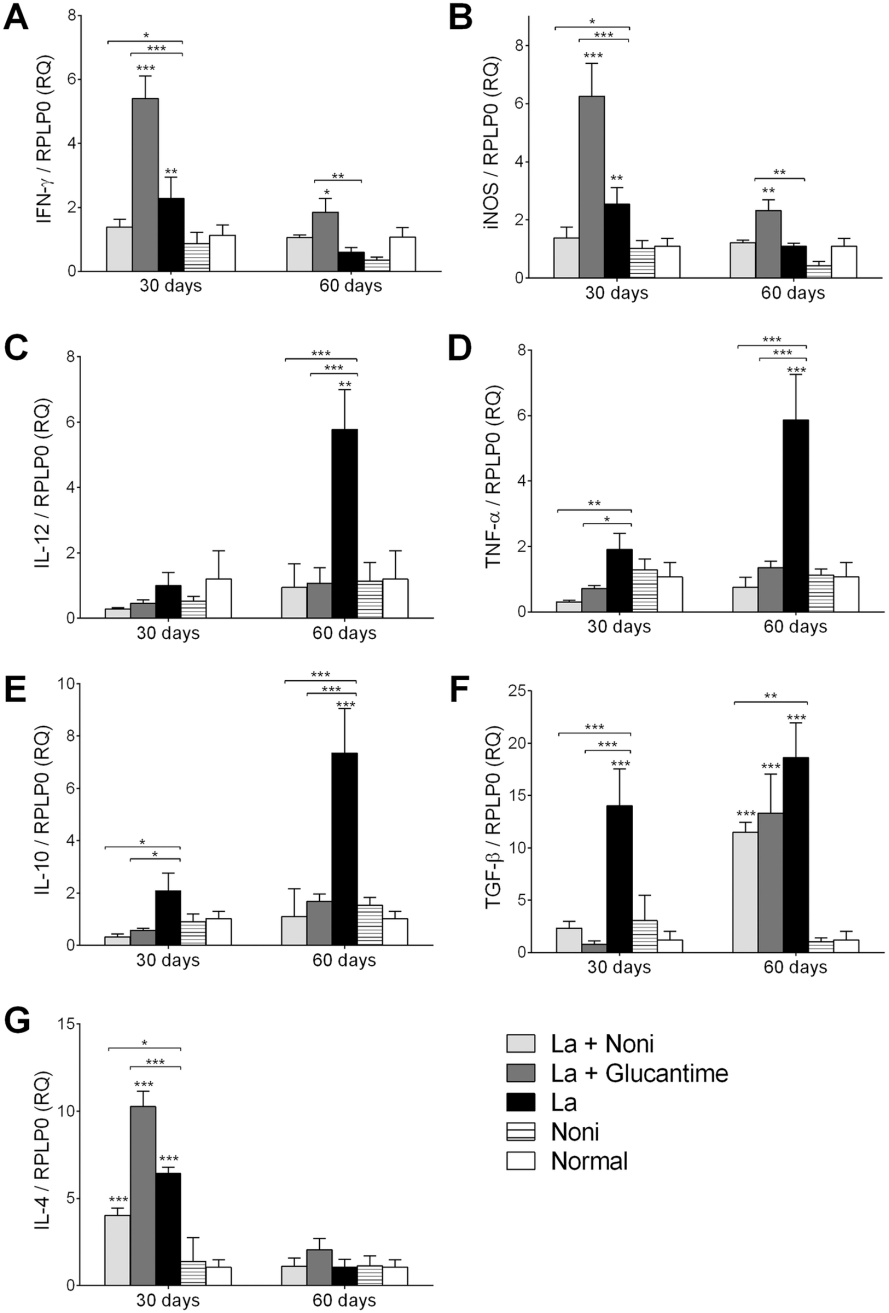
Cytokine gene expression in C57BL/6 mouse footpads infected with *Leishmania (L*.*) amazonensis* and treated with *Morinda citrifolia* fruit juice. Relative quantification of IFN-γ (A), iNOS (B), IL-12 (C), TNF-α (D), IL-10 (E), TGF-β (F) and IL-4 (G) with RPLP0 as endogenous control. Noni group treated at 500mg.kg^-1^.day^-1^ and Glucantime group treated at 20mg.kg^-1^.twice a week^-1^, both during 60 days. Data represent mean ± SD of two experiments realized in triplicate. *p<0.05, **p<0.01, ***p<0.001 when compared with control group or between group brackets by two-way ANOVA and Bonferroni’s post-test. RQ: relative quantification; RPLP0: ribosomal protein large P0; La+Noni: group infected and treated with Noni; La+Glucantime: group infected and treated with Glucantime; La: group infected and mock-treated; Noni: group mock-infected and treated with Noni; Normal: mock-infected and mock-treated group.

When the expressions of IFN-*γ*, iNOS, IL-12, TNF-*α*, IL-10, TGF-*β* and IL-4 at the lesion site were evaluated, no difference was noted between Noni and mock-treated non-infected mice (Fig 4). In mock treated infected mice, IFN-*γ* and iNOS showed upregulation after 30 days of treatment but this was not observed on the 60^th^ day. In Noni treated infected mice this upregulation was not observed and mice showed the normal value throughout the experiment. However, Glucantime treated infected mice presented an upregulation of these cytokines principally on the 30^th^ day of treatment. An upregulation of IL-4 was also noted at this time in Glucantime and mock treated infected mice. The IL-12, TNF-*α* and IL-10 expressions were upregulated in mock treated infected mice especially after the 60^th^ day. This upregulation was not observed in infected and Noni or Glucantime treated mice. TGF-*β* was upregulated throughout the experiment in mock treated infected mice. However, TGF-*β* was upregulated only after 60 days of treatment in Noni or Glucantime treated mice.

**Fig 5:**
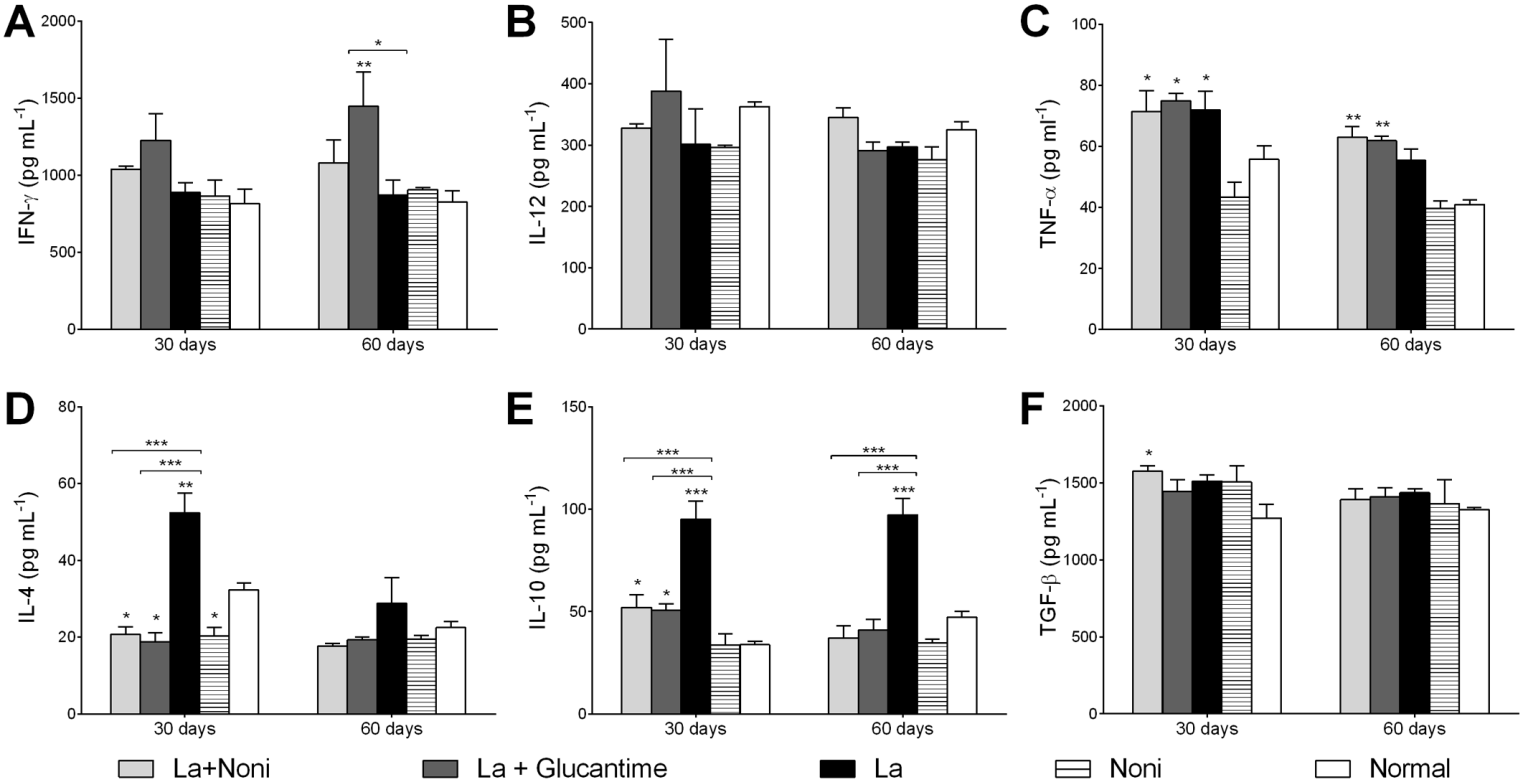
Quantification of serum cytokines from C57BL/6 mice infected with *Leishmania (L*.*) amazonensis* and treated with *Morinda citrifolia* fruit juice. Cytokines levels of IFN-γ (A), IL-12 (B), TNF-α (C), IL-4 (D), IL-10 (E) and TGF-β (F). Noni group treated at 500mg.kg^-1^.day^-1^ and Glucantime group treated at 20mg.kg^-1^.twice a week^-1^, both for 60 days. Data represent mean ± SD of two experiments realized in duplicate. *p<0.05, ***p<0.001 when compared with control group or between group brackets by two-way ANOVA and Bonferroni’s post-test. La+Noni: group infected and treated with Noni; La+Glucantime: group infected and treated with Glucantime; La: group infected and mock-treated; Noni: group mock-infected and treated with Noni; Normal: mock-infected and mock-treated group.

Cytokine levels in the serum showed that *L*. *(L*.*) amazonensis* increased IL-4 and TNF-*α* at 30 days and IL-10 at both treatment times (Fig 5). This high production of TNF-*α* was also observed in the infected groups treated with Noni or Glucantime. IL-10 showed a lower increase in infected and treated groups. On the other hand, IL-4 production decreased in treated groups whether infected or not. No alterations were observed in IL-12 or TGF-*β* except for a slight increase in TGF-*β* production for the Noni treated infected mice. Finally, Glucantime treatment increased IFN production after 60 days.

### Noni enhances extracellular matrix protein

**Fig 6:**
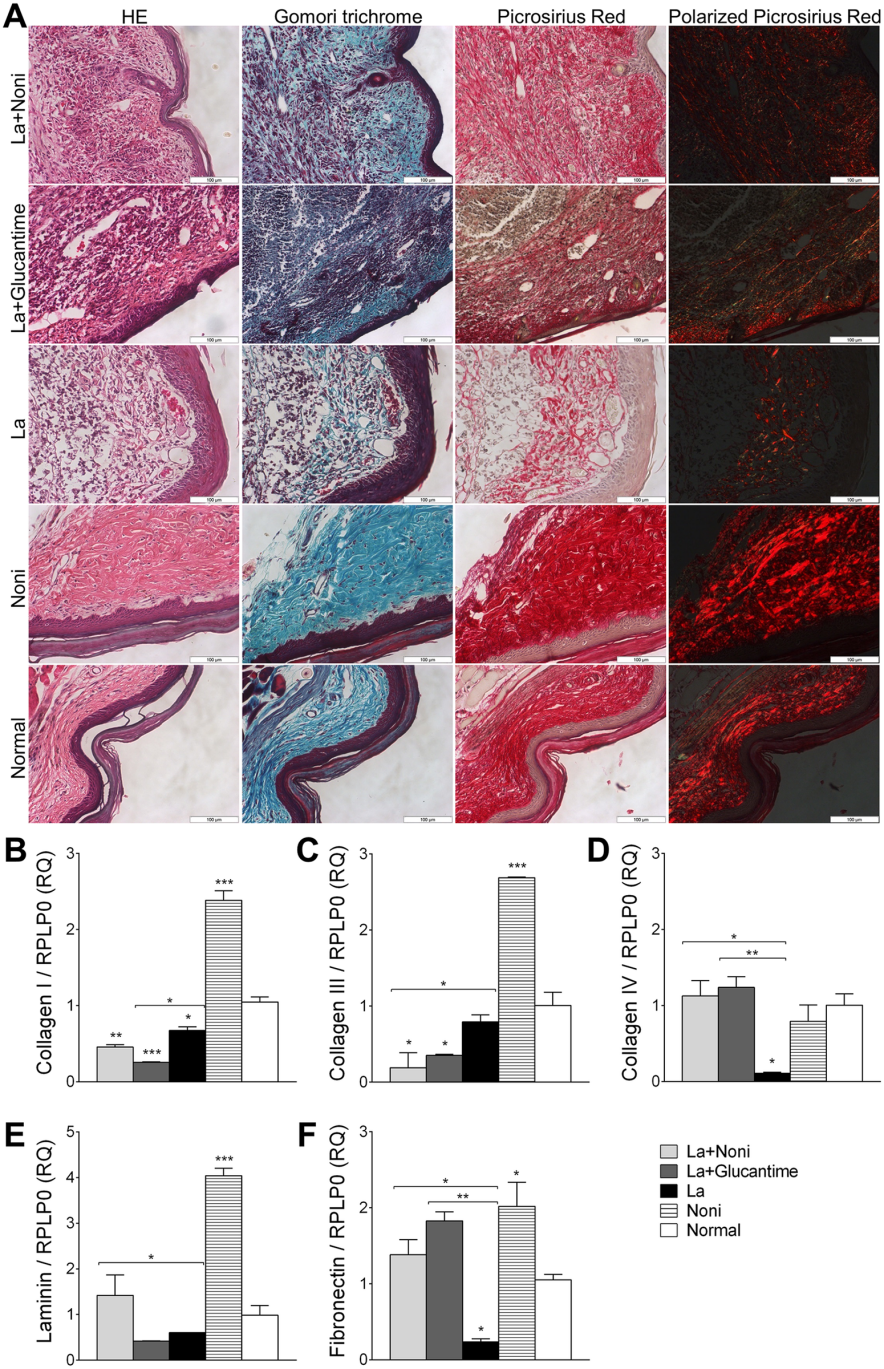
Extracellular matrix protein analysis of C57BL/6 mice footpads infected with *Leishmania amazonensis* and treated for 60 days with *Morinda citrifolia* fruit juice, Noni. Noni group treated at 500mg.kg^-1^.day^-1^ and Glucantime group treated at 20mg.kg^-1^.twice a week^-1^. Histopathology (A) and extracellular matrix protein gene expressions (B-F) of skin. Images are representative of two independent experiments realized in triplicate. HE: hematoxylin-eosin. Data represent mean ± SD of two independent experiments realized in triplicate. *p<0.05, **p<0.01, ***p<0.001 when compared with control group or between group brackets by one-way ANOVA and Bonferroni’s post-test. RQ: relative quantification; RPLP0: ribosomal protein large P0; La+Noni: group infected and treated with Noni; La+Glucantime: group infected and treated with Glucantime; La: group infected and mock-treated; Noni: group mock-infected and treated with Noni; Normal: mock-infected and mock-treated group.

Observation of the lesion site skin stained with hematoxylin-eosin showed a reduction in the normal structure of the dermis and a degradation of the connective tissue in infected footpads when compared with mock-infected groups. This difference in the presence of collagen fibers among the groups was demonstrated using Gomori´s trichrome and Picrosirius Red (Fig 6A). To quantify these alterations, the extracellular matrix protein expression was evaluated by qPCR in footpad skin after 60 days of treatment (Fig 6B–6F). Noni fruit juice upregulated the expression of all analyzed proteins, except for collagen IV in mock-infected mice; whereas *L*. *(L*.*) amazonensis* downregulated the expression of fibronectin, collagen I and IV when compared with normal mice. Noni and Glucantime treatment preserved the normal expression of collagen IV, laminin and fibronectin in infected footpads but they decreased collagen I and III expression.

### Noni showed no toxicity in vivo

**Fig 7:**
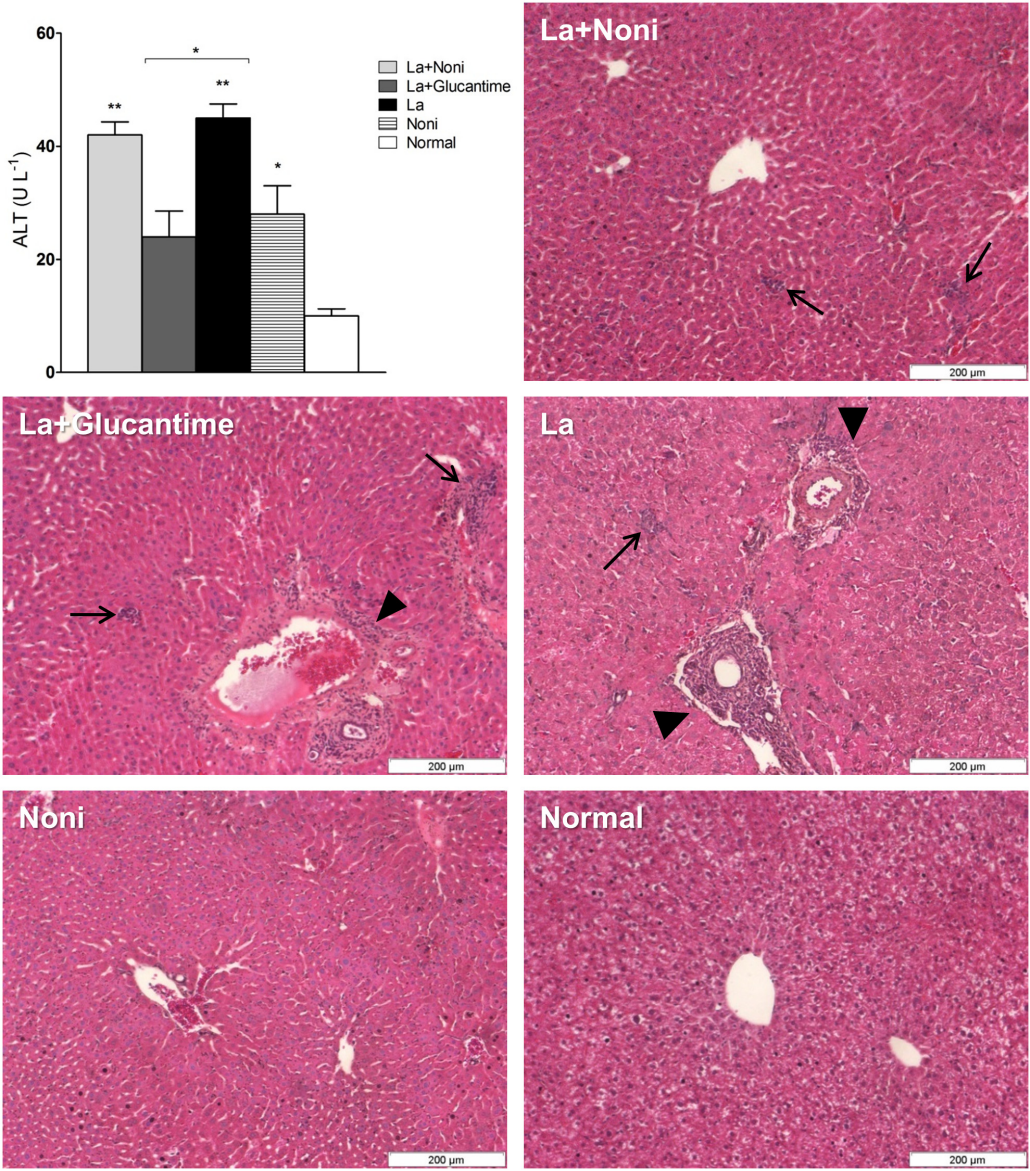
Quantification of alanine aminotransferase (ALT) and histopathology of liver from C57BL/6 mice infected with *Leishmania amazonensis* and treated for 60 days with *Morinda citrifolia* fruit juice, Noni. Noni group treated at 500mg.kg^-1^.day^-1^ and Glucantime group treated at 20mg.kg^-1^.twice a week^-1^. Diffuse inflammatory infiltration (arrows) and periportal infiltration (arrow-heads) in the liver of infected mice. The inflammatory intensity of infiltration decreases with Glucantime and Noni treatments. Hematoxylin-eosin. Data represent mean ± SD of two independent experiments realized in duplicate. *p<0.05, **p<0.01 when compared with control group or between group brackets by one-way ANOVA and Bonferroni’s post-test. La+Noni: group infected and treated with Noni; La+Glucantime: group infected and treated with Glucantime; La: group infected and mock-treated; Noni: group mock-infected and treated with Noni; Normal: mock-infected and mock-treated group.

No clinical signs of toxicity were observed during the treatments and there was no mortality. There was no significant statistical alteration in the weight of the animals after 30 and 60 days of treatment. During necropsy, alterations such as hyperemia were not observed in the stomach or gut mucosa of the animals treated with Noni. Also there was no change in the sera biochemical parameters of hepatic and renal functions, except for alanine transaminase (ALT). Infection and Noni treatment enhanced ALT levels but within the normal maximum limit. Histopathology showed that Noni treatment in mock-infected mice did not stimulate an inflammatory reaction in the liver (Fig 7). The *L*. *(L*.*) amazonensis* on the contrary, induced a diffuse and periportal inflammatory infiltration, the latter being reduced by Noni or Glucantime treatments.

## Discussion

*M*. *citrifolia* has various biological actions including leishmanicidal [6] and immunomodulatory activities [12, 13] that have not yet been fully elucidated. The chromatographic analysis of the Noni juice used in our studies showed the same pattern of other Noni juices produced around the world [8]. It is translucent and brown; presents medium viscosity, characteristic odor, pH 3.9 and yielded 6.31% of a highly hygroscopic powder [7].

In this study we used Noni juice to treat C57BL/6 mice infected with *L*. *(L*.*) amazonensis*. We chose to begin the treatment 55 days after infection, when the lesion was well established, in order to better mimic treatment in humans. In fact, when treatment began, all lesions were about 2mm thick.

Noni treatment decreased the lesion size associated with a lower parasite load in the skin and draining lymph nodes after 60 days of treatment. Treatment with the control drug, Glucantime, caused a faster reduction of the parasite load than Noni. However, after 60 days of treatment, Noni had reduced the lesion size more than Glucantime. The lesion size reduction after Noni treatment is associated with a decreased parasite load and control of the inflammatory process caused by *L*. *(L*.*) amazonensis*. The histopathology and cytokine expression analysis showed a reduction in focal inflammation in the skin after Noni treatment with a downregulation of cytokine expressions (IL-12 and TNF-*α*) at 30 and 60 days of treatment.

IFN-*γ* plays a crucial function in controlling the *Leishmania* infection, as has been demonstrated in mice with genetic defects in this molecule and/or its receptor [14]. IFN-*γ* induces parasite elimination by activating both phagocyte oxidase (phox) and iNOS, which is the most effective mechanism of killing intracellular parasites mediated by macrophages [15, 16]. In vitro, our group demonstrated an increase of nitric oxide production and iNOS expression in the peritoneal macrophages infected with *L*. *(L*.*) amazonensis* and treated with Noni [17]. In the present study, the association of high levels of IFN-*γ* and iNOS and decrease of parasite load was observed in Glucantime treatment, but not in Noni treated mice, suggesting a different mechanism of parasite killing in vivo.

Our results demonstrated that normal levels of IL-10 expression in treated groups were associated to low parasite burden, while high levels of IL-10 expression were associated to elevated parasite burden in mock-treated infected mice showing the role of IL-10 in maintaining the infection. The same results have been described in IL-10 knock-out mice which were more competent in controlling *L*. *(L*.*) major* infection than cells from wild type mice [18]. The TGF-*β* expression was also upregulated in infected mice. Proteins secreted by infected macrophages or the promastigote forms of *L*. (*L*.) *infantum chagasi* activate the soluble form of latent TGF-*β* complex favoring the persistence of parasites within infected macrophages through induction of TGF-*β* mediated anti-inflammatory mechanisms [19]. The role of TGF-*β* as a key predictive factor of enhanced susceptibility to the disease was also demonstrated in BALB/c mice immunized with whole antigens of *L*. *(L*.*) amazonensis*. *S*pecies-specific components of vaccine activate TGF-*β* production that predisposes more susceptible individuals to a more aggravated form of the disease [20]. Thus, a low TGF-*β* expression in Noni treated and infected mice contributes to maintain the control of inflammatory infiltrates when compared with infected mock-treated mice.

The phenotype of susceptibility in *L*. *(L*.*) major* infection is clearly associated to high levels of IL-4 and Th2 response [21]. IL-4 reduces iNOS expression and enhances disease progression due to increased survival and growth of *Leishmania* parasites in infected cells [22]. In our work, *L*. *(L*.*) amazonensis* enhanced the IL-4 expression as expected, while treatment with Noni maintained lower levels of IL-4 expression in infected mice.

As the treatment was performed by gavage, the amount of cytokines in sera allows us to verify the immunomodulatory effect of Noni. In addition, *L*. *(L*.*) amazonensis* infection is not limited to the skin. The parasite tends to disseminate to the lymph nodes and can even reach the spleen and liver [23]. The cytokines measured in sera revealed an enhancement of IL-4 and IL-10 caused by *L*. *(L*.*) amazonensis* infection, which were not seen after Noni or Glucantime treatment. The decreased levels of IL-4 and IL-10 contribute to maintain a Th1 response in the treated groups. Furthermore, the increase of IFN-*γ* levels at 60 days due to Glucantime treatment contributes to the effectiveness of the macrophages by iNOS induction in skin and parasite load decrease. Indeed, Noni treatment decreased the IL-4 levels even in mock-infected mice; this may be due to the activation of cannabinoid 2 receptors [12].

Altogether, these results endorse the immunomodulatory effects of Noni. Studies have reported that an immunochemotherapy is more effective than chemotherapy or immunotherapy [24], and our data show that Noni treatment is actually immunochemotherapy.

In addition to cytokine modulation, the skin histopathology analysis showed that Noni helps to control the inflammatory infiltrates and supports an early remodeling process. The tissue repair process is critically important for rapid cure of cutaneous leishmaniasis, as demonstrated in *L*. *(L*.*) major* murine cutaneous leishmaniasis [25], and is associated to reduced IL-10 and increased TNF-*α*, IFN-*γ* [26] and the TGF-*β* pathway [27]. In a non-infected wound, high levels of IL-10 decrease pro-inflammatory mediators and inflammation, normal collagen deposition and restoration of normal dermal architecture [28], whilst TGF-*β* induces immune cell recruitment, promotes matrix protein synthesis while decreasing matrix protein degradation leading to fibrotic tissue formation [29]. In contrast, Noni treatment promotes a control of the inflammatory process which contributes to a favorable ambient for tissue repair. The increase in TGF-β levels after 60 days of treatment with Noni, when compared to 30 days of treatment, may be associated with this tissue repair.

The excessive secretion of pro-inflammatory cytokines and chemokines, as observed in mock-treated infected mice, can recruit and activate additional inflammatory cells and lead to uncontrolled tissue degradation, including new granulation tissue and growth factors, delaying collagen deposition, which impairs the repair process and perpetuates the non-healing condition [30].

The histopathological evaluation of collagen fibers and protein expression in the skin confirmed a modulation of extracellular matrix proteins in Noni-treated mock-infected mice. Anthraquinones were previously identified in our Noni juice [7] and an anthraquinone isolated from Noni fruit has been shown to stimulate collagen type I, the major component of extracellular matrix of the skin in human dermal fibroblasts. Nano-emulsion with this anthraquinone increased the dermal procollagen I in nude mouse skin [31] in the same way as the Noni increased collagen I expression in mock-infected mice. Moreover, the overexpression of collagen III, laminin and fibronectin by Noni treatment is reported here for the first time.

The extracellular matrix protein expressions most affected by *Leishmania* infection were collagen I, collagen IV and fibronectin. The role of collagen I, IV and fibronectin during *Leishmania* infection have been well described in the literature. *L*. *(L*.*) mexicana* binds fibronectin and collagen I to promote adhesion and phagocytosis by macrophages [32, 33]. Degradation of fibronectin and collagen IV by glycoprotein gp63 seems to enhance *L*. *(L*.*) amazonensis* migration. *Leishmania*-degraded fibronectin by surface and secreted leishmanolysin also decreases the production of reactive oxygen intermediates by parasite-infected macrophages and affects the accumulation of intracellular parasites [34, 35]. Treatment with Noni or Glucantime restored the collagen IV and fibronectin expressions to normal levels. This is possibly due to the reduction of parasitic burden and control of the inflammation process with Noni treatment. In addition, Noni treatment also caused an upregulation of laminin expression, a protein related to the degradation and binding of *Leishmania* [36].

Finally, the toxicity parameters analyzed in our model indicated that Noni treatment has no toxic effect on mice. No alterations in the mucosa of stomach or gut were found, showing that the Noni juice does not irritate the digestive system. This result was expected since a previous work described that *M*. *citrifolia* had a preventive effect on gastro-esophageal inflammatory diseases [37]. Although there was a slight increase in ALT, which did not exceed the normal limits, there was a decrease in the hepatic inflammation caused by *L*. *(L*.*) amazonensis*. Nevertheless, Noni toxicity still needs more studies, considering the controversial data in literature that sometimes show toxicity [38, 39], no toxicity [40–42] or even a liver protective effect [43].

The present work has proved the efficacy of Noni juice in reducing the parasite burden and lesion size. In addition, it has shown its modulatory effects on cytokine and extracellular matrix protein expressions. Altogether, Noni treatment has an antileishmanial activity, associated with an immunomodulatory action, which opens a new path to follow in the quest to promote a rapid clinical cure of cutaneous leishmaniasis.

## Supporting Information

S1 Table Sequence primers used for Real Time PCR. (DOCX)

## References

[1] WHO. Control of the Leishmaniases: report of a meeting of the WHO Expert Comitee on the Control of Leishmaniasis, Geneva, Switzerland, 2010. Geneva: World Health Organization, 2010 Contract No.: 949.

[2] Pace D. Leishmaniasis. J Infect. 2014;69 Suppl 1:S10–8. 10.1016/j.jinf.2014.07.016 . 25238669

[3] Freitas-Junior LH, Chatelain E, Kim HA, Siqueira-Neto JL. Visceral leishmaniasis treatment: What do we have, what do we need and how to deliver it? Int J Parasitol Drugs Drug Resist. 2012;2:11–9. 10.1016/j.ijpddr.2012.01.003 24533267 PMC3862432

[4] Basar S, Uhlenhut K, Högger P, Schöne F, Westendorf J. Analgesic and antiinflammatory activity of *Morinda citrifolia* L. (Noni) fruit. Phytother Res. 2010;24(1):38–42. 10.1002/ptr.2863 . 19548275

[5] Kandaswamy D, Venkateshbabu N, Gogulnath D, Kindo AJ. Dentinal tubule disinfection with 2% chlorhexidine gel, propolis, morinda citrifolia juice, 2% povidone iodine, and calcium hydroxide. Int Endod J. 2010;43(5):419–23. 10.1111/j.1365-2591.2010.01696.x . 20518935

[6] Sattar FA, Ahmed F, Ahmed N, Sattar SA, Malghani MA, Choudhary MI. A double-blind, randomized, clinical trial on the antileishmanial activity of a *Morinda citrifolia* (Noni) stem extract and its major constituents. Nat Prod Commun. 2012;7(2):195–6. . 22474954

[7] Almeida-Souza F, Taniwaki NN, Amaral ACF, Souza CSF, Calabrese KS, Abreu-Silva AL. Ultrastructural Changes and Death of *Leishmania infantum* promastigotes Induced by *Morinda citrifolia* Linn. Fruit (Noni) Juice Treatment. J. Evidence-Based Complementary Altern. Med. 2016 (2016) 9 pages. 10.1155/2016/5063540 PMC4893439 27313649

[8] Potterat O, Felten RV, Dalsgaard PW, Hamburger M. Identification of TLC markers and quantification by HPLC-MS of various constituents in noni fruit powder and commercial noni-derived products. J Agric Food Chem. 2007;55(18):7489–94. 10.1021/jf071359a . 17696360

[9] Grimaldi G, Momen H, Naiff RD, McMahonpratt D, Barrett TV. Characterization and classification of leishmanial parasites from humans, wild mammals, and sand flies in the Amazon region of Brazil. American Journal of Tropical Medicine and Hygiene. 1991;44(6):645–61. ISI:A1991GA22900012. 1858968 10.4269/ajtmh.1991.44.645

[10] Schottelius J, Gonçalves da Costa SC. Studies on the relationship between lectin binding carbohydrates and different strains of *Leishmania* from the New World. Memórias do Instituto Oswaldo Cruz. 1982;77(1):19–27. 7144546 10.1590/s0074-02761982000100003

[11] Sambrook J, Russel DW. Molecular Cloning—A laboratory Manual, Volume 1: Cold Spring Harbor Laboratory Press; 2001 2344 p.

[12] Palu AK, Kim AH, West BJ, Deng S, Jensen J, White L. The effects of Morinda citrifolia L. (noni) on the immune system: its molecular mechanisms of action. J Ethnopharmacol. 2008;115(3):502–6. 10.1016/j.jep.2007.10.023 . 18063495

[13] Nayak S, Mengi S. Immunostimulant activity of noni (Morinda citrifolia) on T and B lymphocytes. Pharm Biol. 2010;48(7):724–31. 10.3109/13880200903264434 . 20645768

[14] Swihart K, Fruth U, Messmer N, Hug K, Behin R, Huang S, et al Mice from a genetically resistant background lacking the interferon gamma receptor are susceptible to infection with *Leishmania major* but mount a polarized T helper cell 1-type CD4+ T cell response. J Exp Med. 1995;181(3):961–71. 7869054 10.1084/jem.181.3.961 PMC2191906

[15] Murray HW, Nathan CF. Macrophage microbicidal mechanisms in vivo: reactive nitrogen versus oxygen intermediates in the killing of intracellular visceral Leishmania donovani. J Exp Med. 1999;189(4):741–6. 9989990 10.1084/jem.189.4.741 PMC2192937

[16] Murray HW, Xiang Z, Ma X. Responses to Leishmania donovani in mice deficient in both phagocyte oxidase and inducible nitric oxide synthase. Am J Trop Med Hyg. 2006;74(6):1013–5. . 16760512

[17] Almeida-Souza F, de Souza CSF, Taniwaki NN, Silva JJ, de Oliveira RM, Abreu-Silva AL, Calabrese KS. Morinda citrifolia Linn. fruit (Noni) juice induces an increase in NO production and death of Leishmania amazonensis amastigotes in peritoneal macrophages from BALB/c. Nitric Oxide. 2016 6 18;58:51–58. 10.1016/j.niox.2016.06.004 27328771

[18] Arcanjo AF, LaRocque-de-Freitas IF, Rocha JD, Zamith D, Costa-da-Silva AC, Nunes MP, et al The PGE2/IL-10 Axis Determines Susceptibility of B-1 Cell-Derived Phagocytes (B-1CDP) to *Leishmania major* Infection. PLoS One. 2015;10(5):e0124888 10.1371/journal.pone.0124888 25933287 PMC4416734

[19] Clemente AM, Severini C, Castronovo G, Tanturli M, Perissi E, Cozzolino F, et al Effects of soluble extracts from *Leishmania infantum* promastigotes, *Toxoplasma gondii* tachyzoites on TGF-β mediated pathways in activated CD4+ T lymphocytes. Microbes Infect. 2014;16(9):778–87. 10.1016/j.micinf.2014.08.002 . 25130316

[20] Pinheiro RO, Pinto EF, Lopes JRC, Guedes HLM, Fentanes RF, Rossi-Bergmann B. TGF-beta-associated enhanced susceptibility to leishmaniasis following intramuscular vaccination of mice with *Leishmania amazonensis* antigens. Microbes and Infection. 2005;7(13):1317–23. 10.1016/j.micinf.2005.04.016. ISI:000233451900004. 16027022

[21] Kopf M, Brombacher F, Köhler G, Kienzle G, Widmann KH, Lefrang K, et al IL-4-deficient Balb/c mice resist infection with *Leishmania major*. J Exp Med. 1996;184(3):1127–36. 9064329 10.1084/jem.184.3.1127 PMC2192785

[22] Hurdayal R, Nieuwenhuizen NE, Revaz-Breton M, Smith L, Hoving JC, Parihar SP, et al Deletion of IL-4 receptor alpha on dendritic cells renders BALB/c mice hypersusceptible to *Leishmania major* infection. PLoS Pathog. 2013;9(10):e1003699 10.1371/journal.ppat.1003699 24204259 PMC3812013

[23] Cardoso FO, de Souza CSF, Mendes VG, Abreu-Silva AL, da Costa SCG, Calabrese KS. Immunopathological studies of *Leishmania amazonensis* infection in resistant and in susceptible mice. Journal of Infectious Diseases. 2010;201(12):1933–40. ISI:000277687900020. 10.1086/652870 20462353

[24] Joshi J, Malla N, Kaur S. A comparative evaluation of efficacy of chemotherapy, immunotherapy and immunochemotherapy in visceral leishmaniasis-an experimental study. Parasitol Int. 2014;63(4):612–20. 10.1016/j.parint.2014.04.002 . 24747611

[25] Sakthianandeswaren A, Elso CM, Simpson K, Curtis JM, Kumar B, Speed TP, et al The wound repair response controls outcome to cutaneous leishmaniasis. Proc Natl Acad Sci U S A. 2005;102(43):15551–6. 10.1073/pnas.0505630102 16223880 PMC1266107

[26] Corware K, Harris D, Teo I, Rogers M, Naresh K, Müller I, et al Accelerated healing of cutaneous leishmaniasis in non-healing BALB/c mice using water soluble amphotericin B-polymethacrylic acid. Biomaterials. 2011;32(31):8029–39. 10.1016/j.biomaterials.2011.07.021 21807409 PMC3168736

[27] Castellucci L, Jamieson SE, Almeida L, Oliveira J, Guimarães LH, Lessa M, et al Wound healing genes and susceptibility to cutaneous leishmaniasis in Brazil. Infect Genet Evol. 2012;12(5):1102–10. 10.1016/j.meegid.2012.03.017 22554650 PMC3372530

[28] Peranteau WH, Zhang L, Muvarak N, Badillo AT, Radu A, Zoltick PW, et al IL-10 overexpression decreases inflammatory mediators and promotes regenerative healing in an adult model of scar formation. J Invest Dermatol. 2008;128(7):1852–60. 10.1038/sj.jid.5701232 . 18200061

[29] Goldberg MT, Han YP, Yan C, Shaw MC, Garner WL. TNF-alpha suppresses alpha-smooth muscle actin expression in human dermal fibroblasts: an implication for abnormal wound healing. J Invest Dermatol. 2007;127(11):2645–55. 10.1038/sj.jid.5700890 17554369 PMC2366884

[30] Rajan V, Murray RZ. The duplicitous nature of inflammation in wound repair. Wound Practice and Research. 2008;16:7.

[31] Kim SW, Jo BK, Jeong JH, Choi SU, Hwang YI. Induction of extracellular matrix synthesis in normal human fibroblasts by anthraquinone isolated from *Morinda citrifolia* (Noni) fruit. J Med Food. 2005;8(4):552–5. 10.1089/jmf.2005.8.552 . 16379572

[32] Wyler DJ, Sypek JP, McDonald JA. In vitro parasite-monocyte interactions in human leishmaniasis: possible role of fibronectin in parasite attachment. Infect Immun. 1985;49(2):305–11. 3160661 10.1128/iai.49.2.305-311.1985 PMC262015

[33] Lira R, Rosales-Encina JL, Argüello C. Leishmania mexicana: binding of promastigotes to type I collagen. Exp Parasitol. 1997;85(2):149–57. 10.1006/expr.1996.4127 . 9030665

[34] Kulkarni MM, Jones EA, McMaster WR, McGwire BS. Fibronectin binding and proteolytic degradation by *Leishmania* and effects on macrophage activation. Infect Immun. 2008;76(4):1738–47. 10.1128/IAI.01274-07 18212076 PMC2292850

[35] McGwire BS, Chang KP, Engman DM. Migration through the extracellular matrix by the parasitic protozoan *Leishmania* is enhanced by surface metalloprotease gp63. Infect Immun. 2003;71(2):1008–10. 12540585 10.1128/IAI.71.2.1008-1010.2003 PMC145380

[36] Ghosh A, Kole L, Bandyopadhyay K, Sarkar K, Das PK. Evidence of a laminin binding protein on the surface of *Leishmania donovani*. Biochem Biophys Res Commun. 1996;226(1):101–6. 10.1006/bbrc.1996.1317 . 8806598

[37] Mahattanadul S, Ridtitid W, Nima S, Phdoongsombut N, Ratanasuwon P, Kasiwong S. Effects of Morinda citrifolia aqueous fruit extract and its biomarker scopoletin on reflux esophagitis and gastric ulcer in rats. J Ethnopharmacol. 2011;134(2):243–50. 10.1016/j.jep.2010.12.004 . 21163341

[38] Millonig G, Stadlmann S, Vogel W. Herbal hepatotoxicity: acute hepatitis caused by a Noni preparation (*Morinda citrifolia*). Eur J Gastroenterol Hepatol. 2005;17(4):445–7. . 15756098 10.1097/00042737-200504000-00009

[39] Stadlbauer V, Fickert P, Lackner C, Schmerlaib J, Krisper P, Trauner M, et al Hepatotoxicity of NONI juice: report of two cases. World J Gastroenterol. 2005;11(30):4758–60. . 16094725 10.3748/wjg.v11.i30.4758 PMC4615426

[40] West BJ, Jensen CJ, Westendorf J. Noni juice is not hepatotoxic. World J Gastroenterol. 2006;12(22):3616–9. 16773722 10.3748/wjg.v12.i22.3616 PMC4087581

[41] West BJ, White LD, Jensen CJ, Palu AK. A double-blind clinical safety study of noni fruit juice. Pac Health Dialog. 2009;15(2):21–32. . 20443518

[42] Mancebo A, I González Y Arteaga ME González BO Fuentes D Hernández O Correa M. Ensayo de toxicidad a dosis repetidas (28 días) por vía oral del extracto acuoso de *Morinda citrifolia* en ratas Sprague Dawley. Revista de Toxicología. 2002;19:6.

[43] Wang MY, Nowicki D, Anderson G, Jensen J, West B. Liver protective effects of *Morinda citrifolia* (Noni). Plant Foods Hum Nutr. 2008;63(2):59–63. 10.1007/s11130-008-0070-3 18317933 PMC2413119

