# Supplementary material for: Morinda citrifolia Linn. Reduces Parasite Load and Modulates Cytokines and Extracellular Matrix Proteins in C57BL/6 Mice Infected with Leishmania (Leishmania) amazonensis
Source: PLoS Negl Trop Dis. 2016 Aug 31;10(8):e0004900. doi: 10.1371/journal.pntd.0004900 (PMC5006983; doi:10.1371/journal.pntd.0004900)
Supplement: S1 Table — (DOCX) [file pntd.0004900.s001.docx]

Supporting Information 1 – Sequence primers used for Real Time PCR

| Target | Primer sequence | | Sequence source |
| --- | --- | --- | --- |
|  | Forward | Reverse |  |
| kDNA1 | GGGTAGGGGCGTTCTGC | TACACCAACCCCCAGTTTGC | M94088 |
| ACTB^a^ | AGAGGGAAATCGTGCGTGAC | CAATAGTGATGACCTGGCCGT | X03672 |
| IL-4 | TTGAACGAGGTCACAGGAGAAG | AGGACGTTTGGCACATCCA | M29854.1 |
| IL-10 | GATGCCCCAGGCAGAGAA | CACCCAGGGAATTCAAATGC | NM_010548.2 |
| IL-12 | ACAGGGTGATGGGCTATCTGA | TGTGGCAGAGGGCCTTGA | NM_001159424.1 |
| TNF-α | CACAAGATGCTGGGACAGTGA | TCCTTGATGGTGGTGCATGA | NM_013693.2 |
| IFN-γ | TTGGCTTTGCAGCTCTTCCT | TGACTGTGCCGTGGCAGTA | NM_008337.3 |
| TGF-β | GCAGTGGCTGAACCAAGGA | AGCAGTGAGCGCTGAATCG | NM_011577.1 |
| iNOS | TTGTCTGCGGCGATGTCA | GAATTCTCTGCACGGTTTGCA | NM_008713.4 |
| Laminin | GCAGGACGACGACGTCATCT | AAGTCTCGAAGTAACGGTGAGTAGGA | NM_001081171.2 |
| Fibronectin | GTGTAGCACAACTTCCAATTACGAA | GGAATTTCCGCCTCGAGTCT | NM_010233.1 |
| Collagen I | CTTCACCTACAGCACCCTTGTG | TGACTGTCTTGCCCCAAGTTC | NM_007742.3 |
| Collagen III | AAGGCGAATTCAAGGCTGAA | TGTGTTTAGTACAGCCATCCTCTAGAA | NM_009930.2 |
| Collagen IV | ACGGGCCAACGCTTCTTC | CATGATCCCAGTCTTTGAGCTCTA | NM_009932.3 |
| RPLP0^b^ | GCCAGCTCAGAACACTGGTCTA | ATGCCCAAAGCCTGGAAGA | NM_007475.5 |

a:β-actina; b: large ribosomal protein, P0.
